# Understanding the link between single cell and population scale responses of *Escherichia coli* in differing ligand gradients

**DOI:** 10.1016/j.csbj.2015.09.003

**Published:** 2015-10-26

**Authors:** Matthew P. Edgington, Marcus J. Tindall

**Affiliations:** a Department of Mathematics & Statistics, University of Reading, Whiteknights, PO Box 220, Reading RG6 6AX, UK; b Institute for Cardiovascular and Metabolic Research, University of Reading, Whiteknights, PO Box 218, Reading RG6 6AA, UK

**Keywords:** Bacteria, Chemotaxis, *Escherichia coli*, Signalling cascade, Multi-scale, Agent-based modelling

## Abstract

We formulate an agent-based population model of *Escherichia coli* cells which incorporates a description of the chemotaxis signalling cascade at the single cell scale. The model is used to gain insight into the link between the signalling cascade dynamics and the overall population response to differing chemoattractant gradients. Firstly, we consider how the observed variation in total (phosphorylated and unphosphorylated) signalling protein concentration affects the ability of cells to accumulate in differing chemoattractant gradients. Results reveal that a variation in total cell protein concentration between cells may be a mechanism for the survival of cell colonies across a wide range of differing environments. We then study the response of cells in the presence of two different chemoattractants. In doing so we demonstrate that the population scale response depends not on the absolute concentration of each chemoattractant but on the sensitivity of the chemoreceptors to their respective concentrations. Our results show the clear link between single cell features and the overall environment in which cells reside.

## 1 Introduction

The chemotactic behaviour of *Escherichia coli* cells has been an influential research area for many years. In particular, research efforts have focused on both the understanding of how single cells produce a chemotactic response and how a colony of cells migrates in a given environment [1], [2]. Each of these aspects has been studied from both theoretical and experimental viewpoints. Studies at the individual cell scale have sought to elucidate the workings of the intracellular signalling pathways leading to the behaviour of the cell's flagella motors which drive the flagella, thus propelling the cell through its environment. As for the behaviour of cell colonies, studies have mainly aimed at explaining the migration of cells within some pre-defined environment. Whilst there exists a large body of literature investigating both single cell and population level phenomena, there has been relatively little work aimed at understanding how single cell features lead to the observed population scale behaviour.

### 1.1 The single cell response

**Fig. 1:**
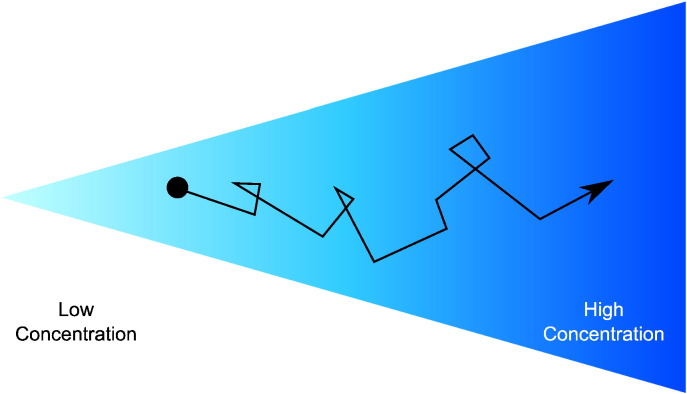
Chemotactic cells utilise a run and tumble swimming pattern in order to find regions containing beneficial nutrients. Runs act to propel the cell forward whereas tumbles act to randomly reorient the cell. When unstimulated, cells execute a three-dimensional random walk, exploring their environment. Upon sensing a beneficial attractant gradient, cells elongate their runs, biasing the random walk in the beneficial direction. This differs from the sensing of a negative gradient after which cells will increase the frequency of tumbles.

Unstimulated, chemotactic *E. coli* cells move about their environment by executing a random walk [3]. In particular, cells swim in (approximately) a straight line (run), however these runs are interspersed with abrupt changes in direction (tumbles). This is often referred to as the chemotactic run and tumble swimming pattern (see Fig. 1). In this run and tumble swimming pattern the direction of movement is altered at least once every few seconds [4]. In order to display chemotaxis, cells increase the length of runs when moving up an attractant gradient [5]. *E. coli* cells utilise an intracellular signalling cascade (as described in Section 1 of the Supporting Text) to control the balance between runs and tumbles, which are the result of counterclockwise (CCW) and clockwise (CW) rotation of the cells flagella, respectively. This allows cells to search for environments which are beneficial for their survival.

### 1.2 Population scale modelling approaches

A range of features at the population scale have been studied using continuum and discrete based approaches. Continuum approaches include the use of partial differential equation (PDE) type models to describe the response of a cell population to differing chemoattractants; the widely known Keller–Segel model being but one example [6]. Whilst such models have been used to help understand population level phenomena, they do not yield insight into how these are caused by individual cell behaviour.

Approaches which have sought to link single cell behaviour to population descriptions include stochastic models, equation-free models and agent-based models (ABM). Stochastic models seek to account for the behaviour of individual cells within an attractant gradient by describing key physiological aspects of the cell response. For instance the work of Alt [7] includes a description of cell tumbling and the turning angle distribution which are described probabilisitically. Under certain conditions the model reduces approximately to a Keller–Segel type model.

Equation-free methods describe cell behaviour on the coarse grained population scale as well as incorporating a more detailed description of the individual cell dynamics. Erban & Othmer [8], [9] and Setayeshgar et al. [10] have notably used such methods. In particular, Setayeshgar et al. [10] showed that larger separation between excitation and adaptation times allow the evolution of the cell population to be coarse grained. Erban & Othmer [8], however, incorporated a simplified microscopic model of the *E. coli* chemotaxis signalling pathway into a telegraph process, subsequently showing that the chemotactic response vanishes as the adaptation time tends toward zero. Results were also generalised to higher dimensions. Equation-free methods go some way toward bridging the gap between single cell and population scale behaviour and greatly help to reduce computational overheads in simulating large scale cell dynamics [9]. However, this is at the expense of being able to elucidate between individual cell behaviour and providing a full description of the underlying cell signalling cascade dynamics.

ABM models are computational in nature and utilise a set of “rules” that allow the effects of single cell attributes to be extrapolated to the population scale. One example is that of Emonet et al. [11] which sought to examine how stochasticity in the chemotactic signalling network impacts upon population level behaviour. In doing so, this model was shown to reproduce a number of features observed in the experimental literature. The model does not, however, include a full description of the individual cell components responsible for chemotaxis, for instance the relationship between CheY-P concentration and flagellar rotational behaviour.

Vladimirov et al. [12] considered an ABM type model that combined a simplified model of the chemotaxis signalling pathway with a detailed description of cell swimming behaviour. In particular, this work showed that varying the concentrations of CheB and CheR proteins (those involved in adaptation) affect the accumulation of cells within different ligand gradients. In particular, they note that cells with too little CheB and CheR tend toward running and thus fail to respond to different ligand gradients.

Bray and colleagues have developed a number of ABMs that provide a good level of agreement with experimental data. In contrast to Emonet et al. [11] and Vladimirov et al. [12], these models aim to capture a greater level of individual cell detail by incorporating a highly detailed (~ 90 ordinary differential equation (ODE)) model of the *E. coli* chemotaxis signalling cascade [13], [14]. This work investigated the impact of cell signalling cascade mutations on cell behaviour. In particular, it was shown that the deletion of CheB and CheR (the proteins responsible for adaptation) resulted in cells that fail to accumulate about greater attractant concentrations.

### 1.3 Multiple ligand detection

**Fig. 2:**
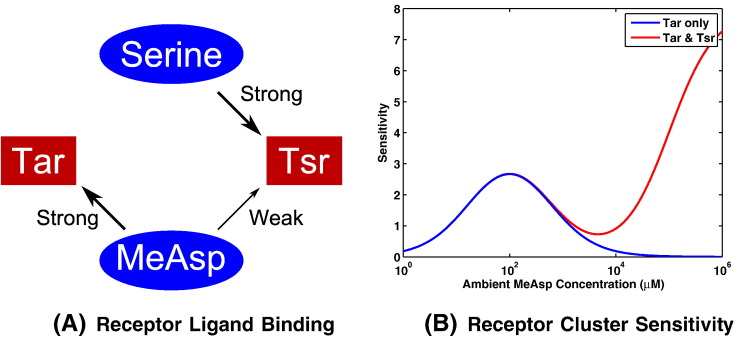
MeAsp and serine bind with strong affinity to Tar and Tsr chemoreceptors, respectively. Additionally, MeAsp is able to bind Tsr chemoreceptors with a low affinity, as seen in panel (A). Panel (B) is reproduced from the work of Mello & Tu [15] and illustrates the difference in the sensitivity of a receptor complex when MeAsp may bind only to Tar receptors (blue) and where the low affinity binding of MeAsp to Tsr chemoreceptors is considered (red). In this work, the low affinity binding of MeAsp to Tsr chemoreceptors is neglected due to the chemoattractant concentrations considered. Note that sensitivity is defined as *S*≡ − ∂lnΦ/∂ ln[*L*], where *S* denotes the sensitivity, Φ is the receptor signalling team activity and [*L*] represents the ligand concentration. (For interpretation of the references to colour in this figure legend, the reader is referred to the web version of this article.)

*E. coli* receptor clusters contain up to five different receptor types which are able to respond to a range of chemoattractants. Tar and Tsr are the two most abundant, which respond to methyl–aspartate (MeAsp) in the case of Tar and Tsr, and serine, in the case of Tsr. However, the Tsr response to MeAsp is neglible for small to intermediate attractant concentrations. Receptor sensitivity to such attractants has been an active area of research which has demonstrated the clear link between receptor occupation and thus sensitivity to differing ligand concentrations [15] as shown in Fig. 2(b).

Within the theoretical literature, it is often assumed that cells respond to just one chemoattractant. This simplifying assumption has clear benefits within a theoretical framework, however more biologically representative is the study of cells when multiple chemoattractants are present.

Here we use an ABM framework to understand the link between the design and dynamics of the cell signalling cascade and the external environment to which cells respond. The model incorporates an ODE model describing the signalling network at the single cell scale [16] as detailed in Section 2 of the Supporting Text. Our ABM is used to explore two aspects of the cellular response in connection with the surrounding environment.

Firstly, it is known experimentally that the total (phosphorylated and unphosphorylated) concentration of intracellular signalling proteins may vary up to ten-fold [17]. Such variation can be due to noise in gene expression and uneven distribution of proteins upon cell division [18]. In fact, Bai et al. [19] have suggested that the expression of key signalling proteins can cause both temporal fluctuations and heterogeneity in the rotational bias of an individual cell's flagellar motors. Thus, we consider how such variation affects the single cell response and how this links to the population scale in differing gradients. We then move to investigate how cells respond in the presence of two spatially distinct gradients of MeAsp and serine.

## 2 Materials and methods

In this section we provide an overview of our ABM algorithm.

### 2.1 ABM Algorithm

The ABM formulated here contains a description of

1. external ligand detection by the cell;
2. the chemotactic signalling pathway described in Section 2 of the Supporting Text;
3 the flagellar rotational bias and response; and
4. the movement of a cell via run or tumble type movement.

Combining these aspects allows us to extrapolate from the single cell to *n* population scale, where *n* is the number of cells in the population. The algorithm is composed of five main stages that proceed in a cyclical manner over a given time period denoted *T*_*s*_. At each time step *t*_*i*_ (*i* ∈ [1, *p*], where *p* = *T*_*s*_/*t*_*i*_):

1. calculate the ligand concentration (at the cell location);
2. update the intracellular signalling pathway;
3. calculate the flagellar rotational bias;
4. simulate cell movement — straight swim (run) or turn and swim (tumble); and
5. return to 1.

**Fig. 3:**
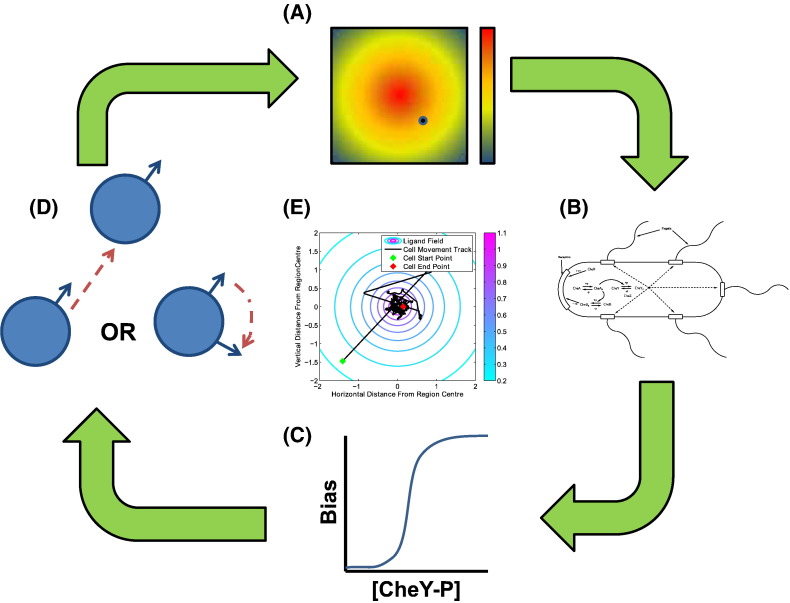
A cartoon diagram showing the workings of our ABM. (A) An initial location is chosen for a simulated *E. coli* cell within a static ligand field. (B) Cells detect the external ligand concentration and responds via an ODE model of the intracellular signalling pathway. (C) The rotational bias of the simulated cells flagella is calculated and a (uniformly distributed) random number generator used to choose a “run” or a “tumble” response. (D) A new location is defined if the cell “runs” or a new direction of travel and a new location are chosen if the cell “tumbles”. The new ligand concentration is calculated and the process repeated for the desired number of time steps, producing results shown in (E).

A graphical summary of the algorithm is given in Fig. 3. The details of ABM Stages 1–4 alongside their respective modelling assumptions and simplifications are examined in more detail in 2.2, 2.3, 2.4. In these simulations each cell is assigned random values for both their initial location within the domain of interest and their initial direction of travel.

Within this work we conduct all simulations using a two-dimensional square spatial domain; a common choice within ABM studies of chemotaxis [11], [13] as it allows for simple interpretation of results. The size of this domain was chosen to be arbitrarily large, and is described by

(1) $$\left(x,y\right)=\left\{x,y\in R:\left[-2,2\right]\right\}\text{,}$$

where *x* and *y* are the horizontal and vertical Cartesian coordinates, respectively.

The behaviour of each cell is then simulated for 50,000 model time-steps, equating to approximately 12 real-time minutes (long enough for > 95% of simulations (using the base parameter set in Table S1) to reach an approximate equilibrium).

### 2.2 Ligand field

A number of different ligand profiles have been studied within both the experimental and theoretical literature. The most common examples are those with exponential, linear and zero gradient profiles [12], [13].

We consider here a number of simplifying assumptions in respect of the ligand field that allow for either easier computation or a more intuitive understanding of our results. We choose to neglect the effects of ligand metabolism which for MeAsp is valid since it is a non-metabolisable attractant. As such we do not need to consider how cells degrade the ligand. We also choose to consider a stationary ligand profile which does not evolve in time. This assumes that the ligand spatiotemporal variation is small in comparison to cell movement on the timescale of the experiment.

**Fig. 4:**
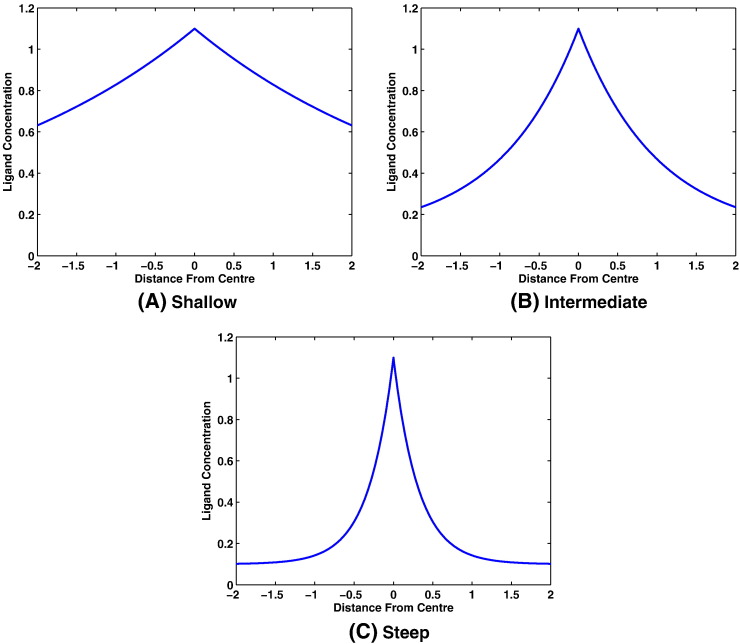
Cross-sectional plots of the three ligand gradients used for agent-based simulations of *E. coli* chemotactic behaviour. Each gradient is of the form shown in Eq. (2) and takes a different value of *d* in order to vary the steepness. Here *d* = 10 produces a shallow gradient (A), *d* = 1 yields an intermediate gradient (B) and *d* = 0.1 gives a steep gradient (C).

Here we focus on the use of exponential ligand gradients of the form

(2) $$\left[L\right]=l_{0}+\exp\left(-\sqrt{\frac{x^{2}+y^{2}}{d}}\right)\text{,}$$

within which [*L*] denotes the ligand concentration (in this case MeAsp), *x* and *y* are the horizontal and vertical Cartesian coordinates of the domain, respectively, *d* is used to vary the steepness of the ligand gradient and *l*_0_ is a minimum ligand concentration (arbitrarily assigned a value of *l*_0_ = 0.1 mM). We consider a shallow gradient (*d* = 10), intermediate gradient (*d* = 1) and a steep gradient (*d* = 0.1) as shown in Fig. 4.

### 2.3 Calculating the cell response

The intracellular signalling cascade ODE model is updated using the inbuilt MATLAB stiff ODE solver (ode15s). This allows us to track the internal state of each simulated cell for every model time-step. As such, we are able to observe the response of CheA–P, CheB–P, CheY–P and the receptor methylation level for each cell over the entire period of an ABM simulation.

**Fig. 5:**
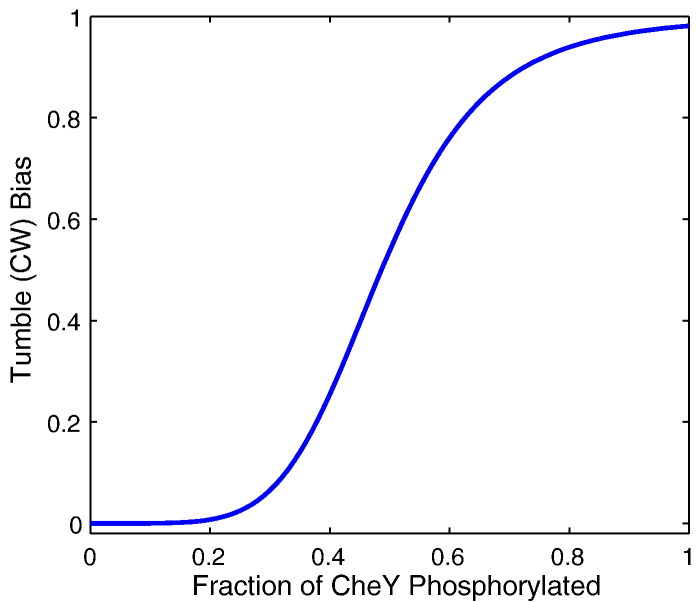
The relationship between CheY–P concentration and the clockwise (CW, tumble) bias of a flagellar motor, as described by Eq. (3). The example shown here is calculated using a value of [*Y*_*p*_]* = 4.043 μM corresponding to the CheY–P steady-state value when utilising the parameter set of Table S1. Note that this curve will shift depending upon the [*Y*_*p*_]* level.

The internal signalling cascade is used to calculate the response of each individual cell. It is known experimentally that the CheY–P concentration acts to regulate the rotational behaviour (bias) of the flagellar motors in *E. coli* cells. There exists two general models of CheY–P and flagellar rotational bias in the literature [20], [21]. The work of Cluzel et al. [20] experimentally quantified this relationship. In doing so it was found that there exists a sigmoidal relationship between CheY–P concentration and CW (clockwise or tumble) bias. This was modelled using a Hill function approach. More recently, Morton-Firth & Bray [21] considered a similar sigmoidal function of the form

(3) $$\mathrm{Bias}\;=\frac{1}{1+\frac{3}{7}{\left(\frac{\left[Y_{p}\right]}{{\left[Y_{p}\right]}^{*}}\right)}^{5.5}}\text{,}$$

where [*Y*_*p*_] is the CheY–P concentration calculated in Section 2.3 and [*Y*_*p*_]* is the concentration in the absence of any stimulus. The resultant sigmoidal curve is displayed in Fig. 5 for the steady-state CheY–P concentration of a wild-type *E. coli* cell. In contrast to the work of Cluzel et al. [20], Eq. (3) allows the sigmoidal curve to shift dependent upon the steady-state CheY–P concentration of each individual cell, allowing the sensitivity of the flagellar response to varying CheY–P concentrations to be modelled.

### 2.4 Simulating cell swimming

In order to accurately represent the swimming behaviour of each simulated cell it is necessary to represent the stochastic nature of flagellar motor switching and the subsequent run and tumble swimming pattern. The ability of *E. coli* cells to produce the observed run and tumble swimming pattern stems from the flagella and the motors controlling their rotation. Explicitly modelling this process would require significant computational cost. Thus, instead we consider a simplified approach that still represents this process to a good degree.

Here we consider the flagellar rotational bias expression from Section 2.3 (i.e. Eq. (3)). This tumble bias denotes the probability that a cell will produce a tumble for any given CheY–P concentration. We therefore utilise a uniformly distributed random number generator to choose a number 0 ≤ *r* ≤ 1 for each simulated cell and assign swimming behaviour according to

$$\mathrm{Flagella}\;\mathrm{direction}=\left\{\begin{array}{ll} CW\left(\mathrm{Tumble}\right), & \mathrm{if}\;\mathrm{Bias}>r\\ CCW\left(\mathrm{Run}\right), & \mathrm{otherwise} \end{array}\right)$$

in which the *Bias* value has been calculated according to Eq. (3). Using this simple approach we represent the stochastic nature of flagellar motor switching without the need for consideration of more complex stochastic equations.

In addition to assigning the type of swimming (run or tumble) behaviour for individual cells, we also consider the resultant movement within the spatial domain described in Section 2.2. During the run phase cells are known to swim in (approximately) a straight line. Mathematically we define this by

(4) $$\frac{dx}{dt}=c\cdot \sin\left(\theta_{n}\right)\text{,}$$

(5) $$\frac{\mathrm{dy}}{dt}=c\cdot \cos\left(\theta_{n}\right)\text{,}$$

where *c* is the swimming speed during a run, *θ*_*n*_ is the angular direction of travel and *x* and *y* are the horizontal and vertical location of the simulated cell in the domain of interest.

During a tumble we also include a turn component, i.e. a change in *θ*_*n*_. This is achieved by considering

(6) $$\theta_{n}=\theta_{o}+\theta_{r}\text{,}$$

within which *n* and *o* are subscripts denoting the new and old values, respectively whilst the subscript *r* indicates a turning angle.

In the case of a run the cell is not re-oriented (*θ*_*r*_ = 0) whereas for a tumble *θ*_*r*_ is chosen according to a uniformly distributed random turning angle of between ± 18 to 98° as per experimental findings summarised in Table 1. Since the duration of a tumble is significantly shorter than that of a run we define a tumble event here as a change in direction from Eq. (6) combined with the movement defined by Eqs. (4), (5).

Consideration must also be given here to the behaviour of cells at the boundary of the spatial domain. Specifically, we require rules governing the behaviour of cells when they pass outside of the spatial domain from Section 2.2. Within the literature there are two main examples considered. These are as follows.

• Periodic: Cells swimming outside of the spatial domain are assumed to re-appear on the opposite side. In the case of the domain in Section 2.2, a cell leaving the domain at (*x*, *y*) = (1, 2) will re-enter the domain at (*x*, *y*) = (1, − 2).
• Solid: Here cells swimming outside of the domain are returned to the boundary as if they swim into a solid wall. For example if, at the end of a given time-step, a simulated cell is positioned at (*x*, *y*) = (2.05, 1) then it will be returned to the boundary at (*x*, *y*) = (2, 1).

In the remainder of this manuscript we consider the solid boundary. This is intended to replicate the behaviour of cells in a bounded region such as a petri dish where they will swim into the solid side wall.

## 3 Applications and results

Here we use our ABM to examine the effect of variation in total intracellular protein concentration on the ability of the population to respond to the ligand fields shown in Fig. 4. Section 3.2 investigates the response of chemotactic cells in the presence of MeAsp and serine.

### 3.1 Effects of variation in intracellular protein concentrations

It has been known for some time that populations of bacterial cells display a significant amount of non-genetic variability [24]. In the context of the *E. coli* chemotaxis signalling pathway the total concentration of each signalling protein (CheA, CheB, CheR, CheW, CheY and CheZ) can vary up to ten-fold between cells [17]. This is known to affect the chemotactic response [25], [26]. In fact, it has been suggested that such variation in the expression of signalling proteins can account for the observed temporal fluctuations and heterogeneity across a population of cells [19]. As such, we use the ABM formulated in Section 2.1 to first investigate how this variation in the total protein concentration affects the individual response before considering the overall population behaviour.

In order to do this we consider a range of different multiples of the total signalling protein concentrations of the form

(7) $${\left[X\right]}_{T}=\beta {\left[X\right]}_{T0}\text{,}$$

where [*X*]_*T*_ (*X* = *A*, *B*, *R*, *Y*, *Z*) represents the total protein concentration of each protein as detailed in Table S1 and *β* (= 1/4, 1/2, 1, 2, 4, 6, 8, 10) denotes its scalar multiple. Here all proteins are scaled together since the operon structure of *E. coli* cells is known to maintain approximately equal ratios between protein concentrations [27].

**Fig. 6:**
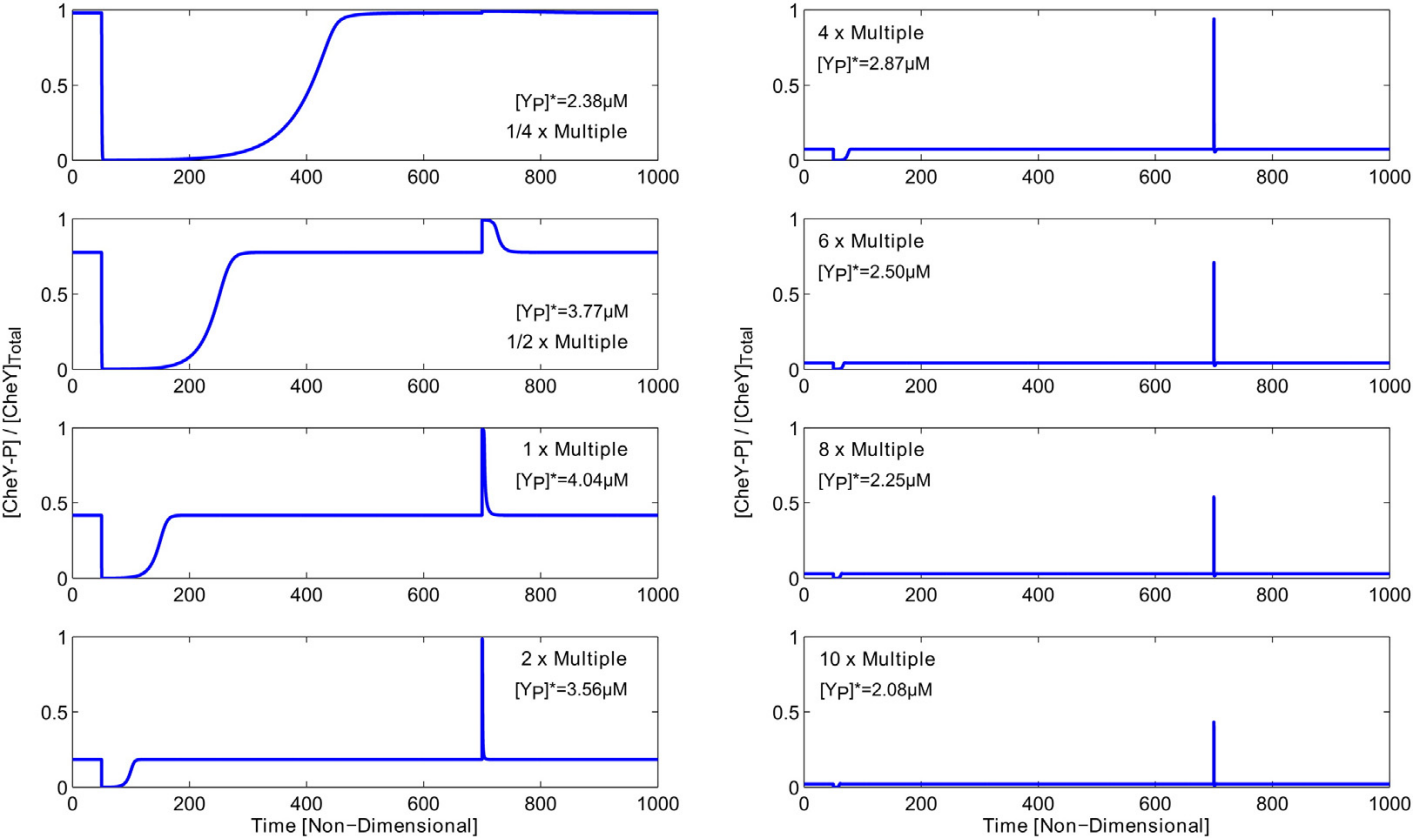
The intracellular signalling response of individual cells to step-changes in the extracellular ligand concentration for varying total protein concentration (*β* = 1/4, 1/2, 1, 2, 4, 6, 8, 10). At non-dimensional time *τ* = 50 the MeAsp concentration is increased from [*L*] = 0.1 mM to [*L*] = 0.2 mM. This is subsequently reversed at *τ* = 700. Dimensional CheY–P steady-state values are given for each case in the respective panels.

Numerical simulations of individual cells (Fig. 6) indicate that larger total protein concentrations lead to:

• lower fractions of phosphorylated proteins at steady-state;
• shorter adaptation times; and
• smaller initial response amplitudes;

in contrast to those with smaller total protein concentrations. These results tend to indicate that the ability to produce long runs is associated with those cells displaying longer adaptation times, i.e. those with smaller total protein concentrations. We may therefore hypothesise that slower adapting cells perform more efficiently in shallower ligand gradients whilst faster adapting ones are more suited to steeper ones.

**Fig. 7:**
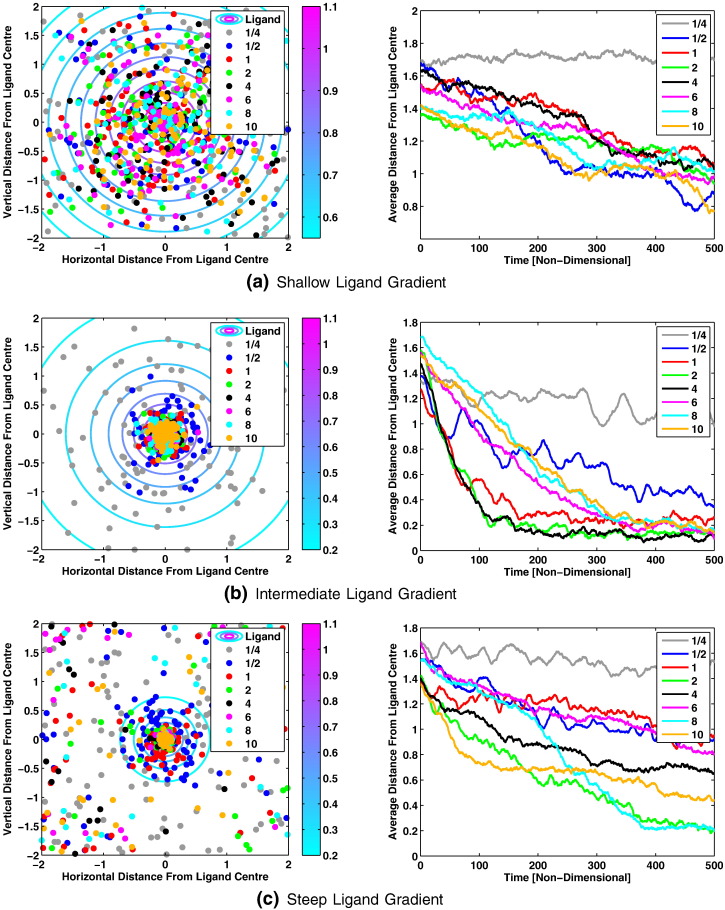
Plots showing (left panel) the final locations of simulated *E. coli* cells and (right) the development in time of the average distance to the peak ligand concentration for each population. Results are shown for; (a) shallow; (b) intermediate; and (c) steep gradients. Each dot (left) refers to the final location of a single cell, whilst (right) lines show the average behaviour of a cell population in time. The different colours denote cell populations with different scaled total protein concentrations, i.e. those with *β* = 1/4 (grey), 1/2 (blue), 1 (red), 2 (green) 4 (black), 6 (pink), 8 (cyan) and 10 (gold). (For interpretation of the references to colour in this figure legend, the reader is referred to the web version of this article.)

To test this hypothesis we simulated the behaviour of 100 individual cells within each of the three ligand gradients shown in Fig. 4. Results obtained from these simulations are displayed in Fig. 7 (see Supplementary Video 1, Supplementary Video 2, Supplementary Video 3 for animations of each simulation).

Upon examination of the results displayed in Fig. 7 a number of interesting features may be observed. In particular, we note that the three ligand gradients considered here result in behavioural differences such as the degree of accumulation, the speed at which accumulation occurs and the range of behaviour observed between cell populations.

**Fig. 8:**
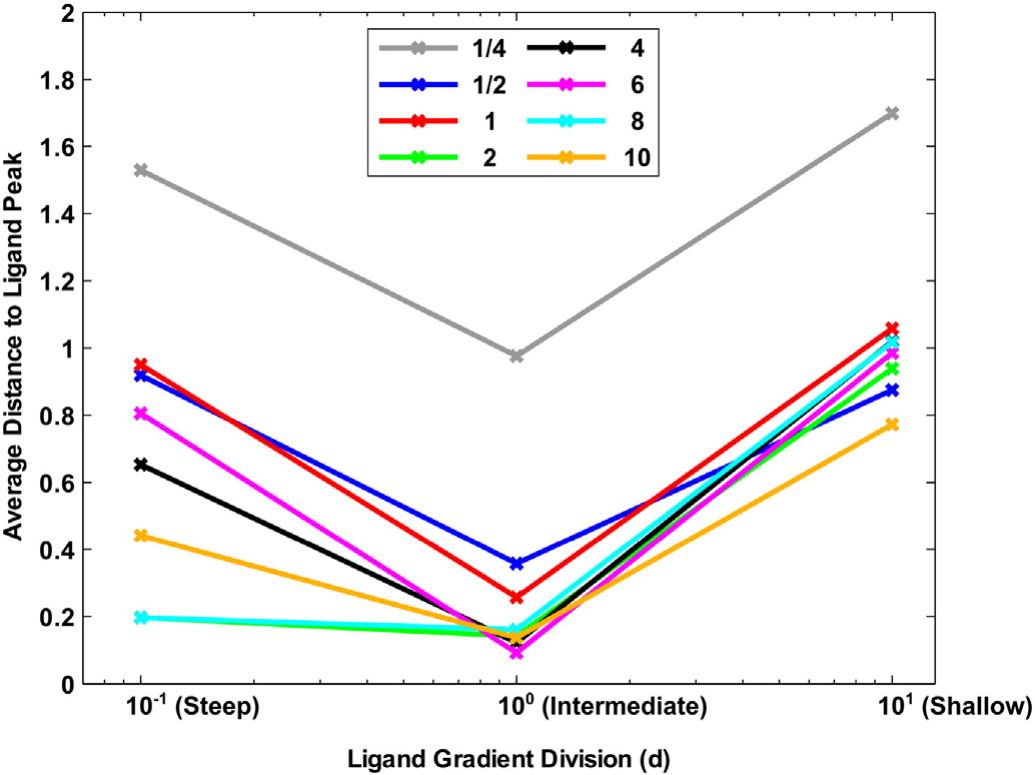
A plot comparing the relative abilities of cell populations with different total protein concentrations to accumulate about the peak of the ligand field for exponential shaped fields of differing steepness. In particular we consider here a steep (left, 10 ^− 1^), intermediate (centre, 10 ^0^) and shallow (right, 10 ^1^) ligand gradient, where the *x*-axis values correspond to *d* in Eq. (2). Coloured lines show the final average distance from the peak ligand concentration achieved by each of the cell populations shown in Fig. 7. The colours of lines indicate the multiples (shown in the figure legend) of all total protein concentrations used in order to create different cell populations.

Firstly, it can be seen from Fig. 7, Fig. 8 that the different gradients result in very different ranges of behaviour. For example, in the shallow ligand gradient all cell populations appear to display a similar degree of accumulation whereas the intermediate and steep gradients display progressively larger differences in accumulation between different cell populations. This suggests that whilst some cell populations may perform better in shallower ligand gradients, the effect is likely to be small in comparison to the differences observed for steeper gradients.

As predicted, the results obtained here indicate that faster adapting cells perform better in steeper ligand gradients. We have already mentioned that behavioural differences associated with the shallow gradient are relatively small across the different populations considered here. As such, we focus our attention more toward the larger differences observed across the intermediate and steep ligand gradients.

From Fig. 8 it can be seen that the three slowest adapting populations (namely *β* = 1/4, *β* = 1/2 and *β* = 1) display relatively poor accumulation in the intermediate gradient compared to the others which all appear to produce similar behaviour. However, upon inspection of Fig. 7(b) it can be seen that cells with intermediate total protein concentrations (i.e. *β* = 2 and *β* = 4) accumulate much faster than those with even shorter adaptation times. Examination of Fig. 8 clearly shows large differences in the final accumulation of different cell populations in the steep ligand gradient. In particular, we notice that cells with longer adaptation times (i.e. *β* = 1/4, *β* = 1/2 and *β* = 1) display the poorest accumulation about the peak ligand concentration. We also note that, as predicted, cells with very fast adaptation times (for example *β* = 8 and *β* = 10) perform well in this case.

The results of Fig. 7, Fig. 8 tend to suggest that, as predicted, cells with faster adaptation times perform better in steep ligand gradients whilst slower adapting cells perform better in shallower ones. There is however an anomaly in that, for the steep ligand gradient, the *β* = 2 population outperforms many with faster adaptation times. To address this point we look to Fig. 4(c) and note that the steep ligand gradient consists of a steep centre portion with much shallower edges. It is these shallow edges that prevent some of the faster adapting cells (that do not perform well in shallow gradients) from performing well in the steep gradient simulation. In particular, we may observe that many fast adapting cells initially struggle to find their way into the steep part of the gradient but then accumulate very rapidly once they do so (see Supplementary Video 3).

In order to explain why cells with shorter adaptation times perform better in steeper ligand gradients we first look to Fig. 6. Here it can be seen that this short adaptation time results in rapid signal termination via CheY–P which leads to a reduction in run time length. Thus the cell runs briefly before tumbling and is optimised for attractant profiles which vary considerably over short spatial intervals. However, in gradients which remain relatively constant, these cells require longer run time lengths in order to seek the optimal attractant concentration.

In the case of *β* = 1/4 we observe very similar behaviour in each of the three gradients considered. This is in agreement with the work of Vladimirov et al. [12] who studied the effect of varying CheB and CheR on the cell response in terms of their final accumulation using an ABM type model. In particular, they note that experimentally and theoretically it can be observed that cells with too little CheB and CheR tend toward running and fail to display tumbles. Within our work we note that at steady-state the *β* = 1/4 cell population almost exclusively displays tumbling behaviour (see Fig. 5, Fig. 6). However, when these cells detect a positive change in ligand concentration their CheY–P concentration falls for a long period due to their very slow rate of adaptation. Thus the cells exhibit very long runs and fail to display effective chemotaxis for the gradients considered (see Fig. 7). This is in agreement with the results of Vladimirov et al. [12].

### 3.2 Chemotaxis in the presence of two attractants

To model the response of cells in our ABM to the attractants MeAsp and serine we first needed to adapt the description of the receptor ligand response. This results in a free-energy expression of the form in equation (S6).

**Fig. 9:**
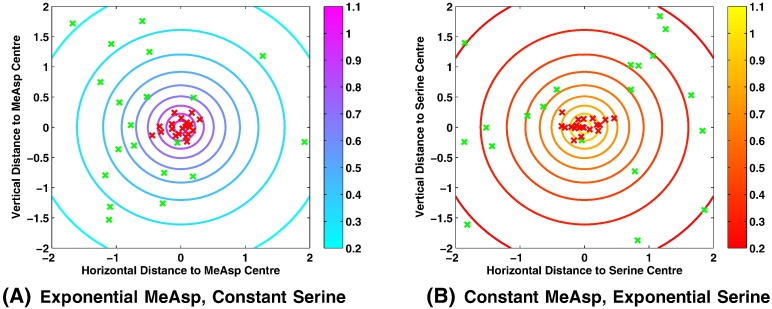
Plots demonstrating the ability of simulated *E. coli* cells to accumulate in response to both MeAsp and serine concentration gradients. Shown here are typical cases in which both MeAsp and serine are present within the same domain. In (A) we consider a constant concentration of serine across the whole domain, thus demonstrating the ability of simulated cells to respond to a MeAsp concentration gradient. In (B) we consider a constant concentration of MeAsp across the entire domain, thus showing that cells are able to respond to a serine concentration gradient. Within each plot green and red crosses indicate the starting and final locations of each simulated cell, respectively whilst contour lines and colour bars show the concentration of the non-constant ligand across the domain. (For interpretation of the references to colour in this figure legend, the reader is referred to the web version of this article.)

To check cells within our model respond both to MeAsp and serine we conducted simulations in which one spatially varied whilst the other was held constant. This gave the result in Fig. 9 and confirmed the model exhibited the expected behaviour.

Using our ABM framework we next examined the population response when two spatially distinct ligand gradients of the form

(8) $$\begin{array}{cc} \left[L_{a}\right]=\omega \left(l_{a0}+\exp\left(-\sqrt{{\left(x+x_{a}\right)}^{2}+y^{2}}\right)\right), & \left(\mathrm{MeAsp}\right) \end{array}$$

(9) $$\begin{array}{cc} L_{s}]=\upsilon \left(l_{s0}+\exp\left(-\sqrt{{\left(x+x_{s}\right)}^{2}+y^{2}}\right)\right), & \left(\mathrm{Serine}\right) \end{array}$$

are present and where [*L*_*a*/*s*_] denotes the concentration of MeAsp or serine, *l*_0_ indicates a minimum chemoattractant concentration, *x* and *y* denote horizontal and vertical coordinates, *ω* and *υ* are scaling parameters. Note that *x*_*a*_ and *x*_*s*_ allow the ligand peaks to be located at different spatial locations in the domain of interest. Here we choose *x*_*a*_ = 1 and *x*_*s*_ = − 1, resulting in an exponential MeAsp gradient centred about (*x*, *y*) = (− 1, 0) and an exponential serine gradient centred about (*x*, *y*) = (1, 0). Note that each ligand gradient is altered via a simple multiplicative scaling. Since *E. coli* cells have been shown to exhibit logarithmic sensing [28], we need not consider the effects of gradient steepness.

**Fig. 10:**
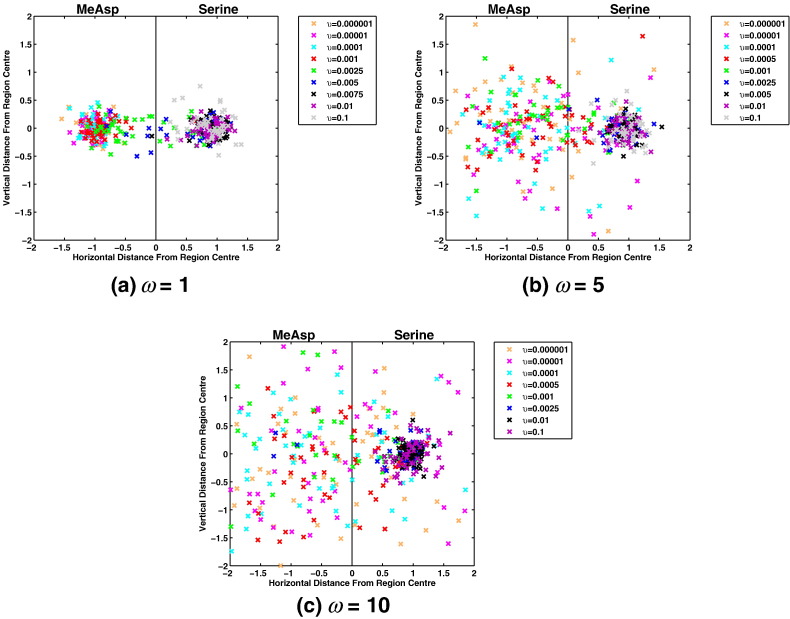
Plots showing the final positions of simulated cells after 50,000 model time-steps (≈ 12 min) for spatially separate MeAsp and serine ligand gradients. In each case the chemotactic response is to a gradient of MeAsp centred in the left half of the domain and a serine gradient centred in the right half of the domain. Coloured crosses show the final position of each simulated *E. coli* cell with the colour indicating the multiple of the serine gradient (i.e. the value of *υ* within Eq. (9)). The three separate panels relate to the multiple applied to the MeAsp gradient (i.e. the ω value in Eq. (8)). Here the peak concentrations for each chemoattractant gradient are given by [*L*_*a*_]_*peak*_ = *ω*(*l*_*a*0_ + 1) for MeAsp and [*L*_*s*_]_*peak*_ = *υ*(*l*_*s*0_ + 1) for serine, where minimum concentrations are chosen such that *l*_*a*0_ = 0.1 mM = *l*_*s*0_. Considered here are values of ω = 1 (top left), ω = 5 (top right) and ω = 10 (bottom).

Using the two scaling parameters (ω and *υ*) it is possible to assess where cells will accumulate under a variety of differences in the concentration of each attractant. In particular we consider three different scalings for the MeAsp gradient, namely ω = 1, ω = 5 and ω = 10. For each of these we considered a range of scalings for various values of *υ* (Table 2). For each pair of ω and *υ* values we conducted a simulation of a population of 50 cells. Results obtained from these simulations are displayed in Fig. 10.

**Fig. 11:**
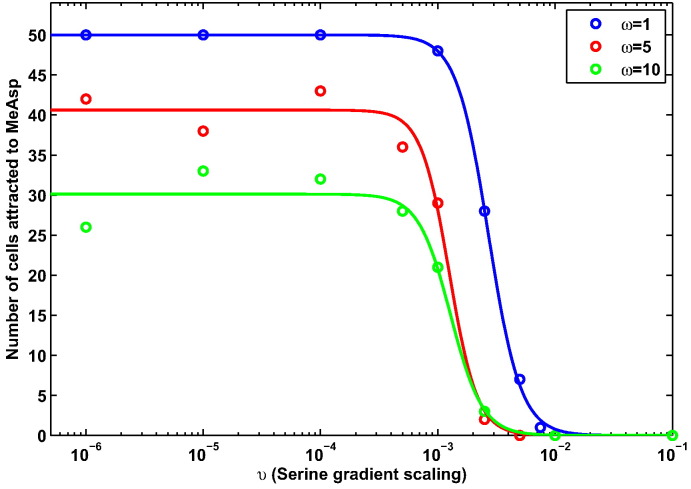
A plot summarising the accumulation of simulated *E. coli* cells toward gradients of MeAsp and serine with differing peak concentrations. Results displayed here represent a summary of those in Fig. 10 with cells considered to accumulate to MeAsp if they end with *x* < 1 and to serine where they finish with *x* > 1. Circles represent the data points drawn from Fig. 10 with the colour indicating the MeAsp gradient scaling factor where ω = 1 (blue), ω = 5 (red) and ω = 10 (green). Since the ABM is stochastic, lines are used to display the general trend of the data. In particular a Hill function is fitted to each set of data using a simple least-squares fit giving values of *K* = 2.71 × 10^− 3^ and *n* = 3.166 for ω = 1; *K* = 1.25 × 10^− 3^ and *n* = 3.605 for ω = 5; and *K* = 1.28 × 10^− 3^ and *n* = 3.180 for *ω* = 10. (For interpretation of the references to colour in this figure legend, the reader is referred to the web version of this article.)

It can be seen in Fig. 10 that there are a number of conditions on ω and *υ* which result in different numbers of cells being attracted to each gradient. Using these ABM simulations we may count the number of cells accumulating toward each attractant. For simplicity we consider a cell to be attracted to MeAsp if the final location is such that *x* < 0. If *x* > 0 we say the cell was attracted to the serine gradient. In order to more clearly elucidate how the relationship between ω and *υ* affects the accumulation of cells about the two different ligands we consider the total number of cells attracted to MeAsp versus serine, as summarised in Fig. 11.

**Fig. 12:**
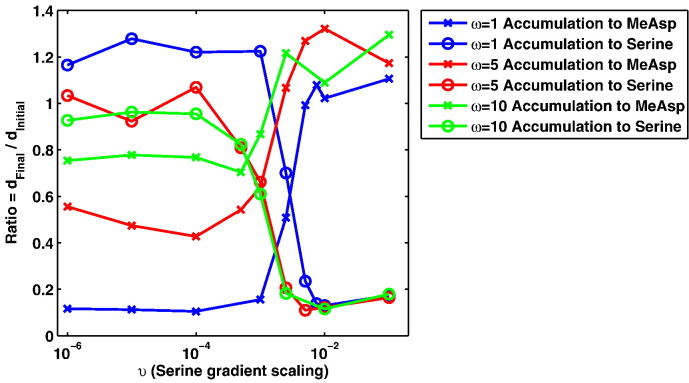
A plot displaying the degree of accumulation exhibited by simulated *E. coli* cells to simultaneously occurring gradients of MeAsp and serine. Results displayed here represent a summary of those in Fig. 10. Here, for each simulation conducted, we plot the ratio between the final and initial average distances from each peak ligand concentration (calculated as Ratio = *d*_*Final*_ /*d*_*Initial*_ where *d*_*Initial*_ and *d*_*Final*_ are the initial and final average distances to a peak ligand concentration, respectively). This is used to give a measure of the extent of accumulation that occurs in each case with a smaller value indicating a greater degree of accumulation. Blue, red and green lines indicate results of simulations conducted using ω = 1, ω = 5 and ω = 10, respectively. Crosses display accumulation toward the attractant MeAsp whereas circles indicate accumulation toward serine. Crosses display accumulation toward the attractant MeAsp whereas circles indicate accumulation toward serine. Note that smaller ratio values indicate a greater degree of accumulation whereas ratio values close to one suggest no accumulation at all. (For interpretation of the references to colour in this figure legend, the reader is referred to the web version of this article.)

It is clear from Fig. 11, Fig. 12 that there is a critical *υ* value below which some cells will begin to be attracted to the MeAsp gradient. It also appears that for larger values of ω this critical value decreases. At first this may appear counter intuitive — why would a greater MeAsp concentration be overcome by a smaller concentration of serine? In order to answer this question we refer to the attractant concentration versus receptor sensitivity curve as described by Mello & Tu [15] and consider this in the context of the ligand concentrations considered here. First note values of ω = 1, ω = 5 and ω = 10 correspond to peak MeAsp concentrations of 1.1 mM, 5.5 mM and 11 mM, respectively. Examining these in the context of the sensitivity curve [15] it can be seen that for the three examples considered here, an ω = 1 scaling produces the greatest sensitivity and ω = 10 (due to saturation of receptors) produces the least (see Fig. 2(b)). This also explains the differences observed in the accumulation of cells to MeAsp in the three gradients considered here. As such, there is clear theoretical support for the idea that increasing the peak concentration of one ligand will not necessarily require increasing amounts of another ligand in order to overcome cells being attracted to it. In fact, upon examining the results of Fig. 10 the receptor sensitivity can be seen to play a role in determining the ability of cells to accumulate about the peak MeAsp concentration. In Fig. 10(a), the example with the greatest sensitivity to MeAsp, it is clear that there is strong accumulation toward the peak concentration located at (*x*, *y*) = (− 1, 0) (demonstrated by a ratio between initial and final distances to the MeAsp peak of ~ 0.15 in Fig. 12). This differs from panel (b) and (c) where the accumulation is clearly less strong, as evidenced by the reduced accumulation about (*x*, *y*) = (− 1, 0). It is in fact possible to observe weak accumulation in panel (b), corresponding to ω = 5 whereas panel (c) (ω = 10) displays virtually no accumulation toward the MeAsp peak concentration (as seen in Fig. 12 where the ratio between the initial and final distances to the peak MeAsp concentration is ~ 1). This strongly suggests that the link between the ability to accumulate toward a certain ligand concentration coupled with receptor sensitivity is causing the emergence of the behaviour observed here.

Upon further consideration of the results here it is clear that the sensitivity of chemoreceptors to MeAsp alone does not quite tell the whole story. It is clear that the sensitivity of chemoreceptors to MeAsp is responsible for the ability of cells to accumulate about a peak MeAsp concentration. This, however, will not directly affect the ability of cells to accumulate in response to a serine chemoattractant gradient apart from the fact that the two chemoreceptor types share a common intracellular signalling pathway in order to produce a single response. In order to consider the ability of cells to accumulate about serine we look to Fig. 11. It is clear from these results that the *υ* value at which cells begin to accumulate toward MeAsp is fairly similar in each of the three examples. This would suggest that a ligand sensitivity curve similar to that for MeAsp is acting to control the sensitivity of the response to serine. In particular, for values of *υ* > 10^− 1^ it is clear that there must be a high level of sensitivity to the serine gradient since all 50 cells in each example are attracted toward the serine peak. For *υ* < 10^− 4^ we would expect a low sensitivity toward the peak aspartate concentration since this is the region in which the fewest cells are attracted to the serine gradient. We should therefore expect that in the range 10^− 4^ < *υ* < 10^− 1^ we should observe a decreasing sensitivity to the serine peak concentration as the value of *υ* is decreased.

## 4 Discussion and conclusions

In this manuscript we have used an ABM to understand the link between the individual *E. coli* chemotactic response and the population scale response in differing attractant environments. Firstly, we investigated the effects of variation in total intracellular signalling proteins on the ability of cell populations to accumulate within different ligand gradients. We then went on to examine the responses of cells to two spatially distinct gradients of MeAsp and serine.

Studying the effects of variation in intracellular signalling protein concentrations revealed significant differences in the ability of cells to accumulate about the peak concentration of a given ligand gradient. More specifically, those cells displaying shorter adaptation times (i.e. those with larger intracellular protein concentrations) performed more effectively in steep ligand gradients whereas those with longer adaptation times were more effective in shallow ones. This is due to the fact that faster adapting cells are better able to deal with sharp changes in ligand concentration, thus ensuring they maintain a beneficial swimming direction whereas cells with longer adaptation times (i.e. cells with smaller signalling protein concentrations) can produce longer runs which are more beneficial in shallower ligand gradients.

Since experimental results have shown a great degree of non-genetic variation, a colony will consist of individual cells each containing different signalling protein concentrations and thus differing chemotactic responses. This is likely to represent a mechanism allowing cell colonies to survive across a wide range of different extracellular environments [19]. For example, cells with large (small) intracellular protein concentrations will be able to survive in environments containing steep (shallow) ligand gradients. It is therefore likely that a colony containing cells with a range of different intracellular protein concentrations will allow a subset of cells to survive within most environments. This surviving subset of cells are then able to divide, thus replacing those cells that have been lost leading to repopulation of the colony. Our results mirror the recent work of Frankel and colleagues [29]. They investigated the role of non-genetic variability and the cellular environment, but greatly simplified the cell signalling cascade, its intuitive biological connection to the cell response physiology and did not consider the various populations individually for differing gradients as done so here.

Whilst the majority of both the experimental and theoretical literature focuses on the ability of cells to form a chemotactic response to one chemoattractant (usually MeAsp), Section 2.3 went a step further and examined how cells respond in the presence of two different chemoattractants. It was shown here that the response of a cell population would be determined by the sensitivity of the chemoreceptors to the precise chemoattractant concentrations present. Cells will accumulate toward a ligand concentration which they are most sensitive to, but which is not necessarily the largest absolute concentration. In the case of two competing gradients it is necessary to compare the sensitivity of cells to each (using expressions such as that described by Mello & Tu [15]) in order to assess which gradient will be preferred.

We postulate here that the ability to respond to two (or possibly more) ligand gradients with varying sensitivities may be advantageous in environments in which mixtures of ligands are present. Here the response to the more sensitive ligand, should it confer more survivability on the cell itself, would not be affected by the overall concentration of ligands within the mixture, thus allowing the cell to ensure its response to important and possibly life-sustaining ligands is maintained.

The results discussed within this manuscript demonstrate some of the potential uses of agent-based modelling in the study of bacterial chemotaxis. In fact, this work suggests that approaches such as that demonstrated here could even help in the study of as yet understudied systems at either the single cell or population scale. ABMs of such systems which, from an experimental perspective, are not fully understood could provide an initial round of model invalidation in which models that do not produce experimentally observed behaviours, at one or both scales, may be identified and rejected more rapidly than may be the case in more conventional single cell studies.

The following are the supplementary material related to this article.

Supplementary material.

Supplementary Video 1 An animation showing the simulated behaviour of differing cell populations within a shallow MeAsp gradient. Note that this is an animation of the results displayed in Figure 7(a).

Supplementary Video 2 An animation showing the simulated behaviour of differing cell populations within an intermediate MeAsp gradient. Note that this is an animation of the results displayed in Figure 7(b).

Supplementary Video 3 An animation showing the simulated behaviour of differing cell populations within a steep MeAsp gradient. Note that this is an animation of the results displayed in Figure 7(c).

## Figures and Tables

**Table 1:** Experimental parameter values describing the swimming behaviour of *E. coli* cells.

Symbol	Definition	Value	Source
*c*	Swimming speed during a run	29 ± 6 μm/s	[22]
*θ*_*r*_	Angle turned during a tumble	58 ± 40°	[23]

**Table 2:** Ligand gradient scalings used for multiple ligand simulations and their respective peak concentrations.

Scaling	Attractant	Peak concentration
ω = 1	MeAsp	1.1 mM
ω = 5	MeAsp	5.5 mM
ω = 10	MeAsp	11 mM
*υ* = 0.000001	Serine	1.1 × 10^− 6^mM
*υ* = 0.00001	Serine	1.1 × 10^− 5^mM
*υ* = 0.0001	Serine	1.1 × 10^− 4^mM
*υ* = 0.0005	Serine	5.5 × 10^− 4^mM
*υ* = 0.001	Serine	1.1 × 10^− 3^mM
*υ* = 0.0025	Serine	2.75 × 10^− 3^mM
*υ* = 0.005	Serine	5.5 × 10^− 3^mM
*υ* = 0.0075	Serine	8.25 × 10^− 3^mM
*υ* = 0.01	Serine	1.1 × 10^− 2^mM
*υ* = 0.1	Serine	1.1 × 10^− 1^mM
