# Supplementary material for: Understanding the link between single cell and population scale responses of Escherichia coli in differing ligand gradients
Source: Comput Struct Biotechnol J. 2015 Oct 26;13:528–38. doi: 10.1016/j.csbj.2015.09.003 (PMC4660157; doi:10.1016/j.csbj.2015.09.003)
Supplement: Supplementary file 1 — Supplementary material. [file mmc1.pdf]

Supporting Text

Linking the Single-Cell and Population-Scale Chemotactic Responses of *Escherichia coli* to Different Ligand Gradients

Matthew P. Edgington[a] and Marcus J. Tindall[a,b]

[a]Department of Mathematics & Statistics, University of Reading, Whiteknights, PO Box 220, Reading, RG6 6AX, UK.
[b]Institute for Cardiovascular and Metabolic Research, University of Reading. PO Box 218, Whiteknights, Reading, RG6 6AA, UK.

1 The Single Cell Chemotactic Response of *Escherichia coli*

The signalling pathway responsible for chemotaxis in *E. coli* is well understood (see Figure S1). This pathway begins at the poles of the cell, where arrays of transmembrane chemoreceptors are located. Molecules of chemoattractants present within the environment are able to bind to the extracellular domains of the receptors affecting their state of activity. This leads to the creation of a signal that is passed into the cell. In order to translate this into a pathway response, the intracellular domains of chemoreceptors associate with a linker protein CheW. A histidine protein kinase (CheA) is then able to bind CheW, thus forming functional chemotaxis complexes that are localised to the poles of the cell [1]. Once these complexes are formed, CheA is able to autophosphorylate (forming CheA-P) at a rate dependent upon the activity of the chemoreceptors.

Phosphoryl groups from CheA-P may then be passed onto either the response regulator protein CheY or the methylesterase CheB [2]. Phosphorylated CheY (CheY-P) then diffuses within the cell cytoplasm toward the flagellar motors [3, 4]. Upon binding of CheY-P to the flagellar motor-switching protein FliM, a greater bias towards tumbling is observed [5, 6]. CheY-P is able to return to its unphosphorylated state (CheY) via autodephosphorylation. Additionally, a phosphatase (CheZ) increases the rate at which this dephosphorylation occurs, leading to more rapid signal termination [7].

An adaptation module functions within *E. coli* cells to re-set chemoreceptors to their pre-stimulus state. This is comprised of phosphorylated CheB (CheB-P) and the methyltransferase CheR. Within this adaptation module, CheR acts to constantly methylate chemoreceptors, increasing their activity [8] whilst CheB-P demethylates chemoreceptors, decreasing their activity [9].

When a positive change in chemoattractant concentration is sensed, the activity of chemoreceptors falls. This results in a lowering of the CheA autophosphorylation rate, thus causing a drop in CheY-P and CheB-P concentrations. The reduced CheY-P concentration causes an increase in the amount of counterclockwise (CCW) flagellar rotation, causing a chemotactic run. The lowered CheB-P concentration allows the constant action of CheR to methylate receptors. This causes an increase in chemoreceptor activity, thus resulting in a return to pre-stimulus CheA-P, CheB-P and CheY-P concentrations.

For a negative chemoattractant change, receptor activity rises as does the rate of CheA autophosphorylation. This leads to a subsequent CheY-P increase, thus causing the flagellar motors to rotate clockwise (CW) causing more regular tumbling. The concentration of CheB-P also rises, causing the demethylation of receptors and a resultant fall in their activity. This causes a return to a pre-stimulus rate of CheA autophosphorylation and in turn CheB-P and CheY-P concentrations.

2 Signalling Pathway Model

Here we summarise a slightly altered version of the Clausznitzer et al. [10] model of *Escherichia coli* chemotaxis signalling used within our agent-based model. Within this section we provide a summary of the

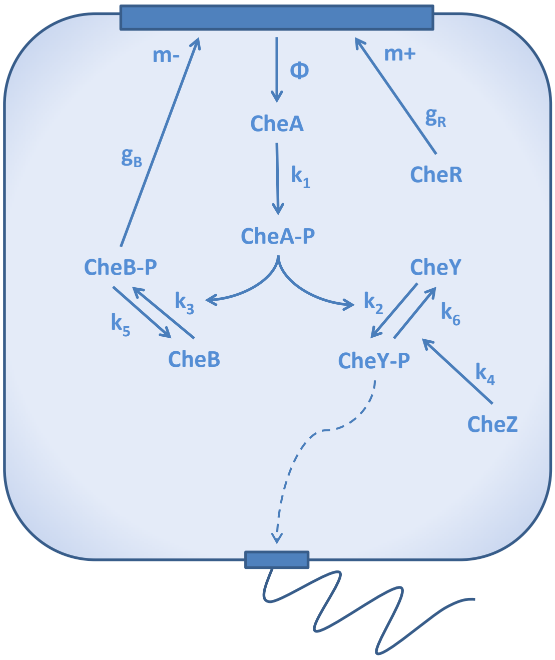

Figure S1: A schematic representation of the intracellular signalling pathway in *E. coli* chemotactic bacteria. Solid lines represent reactions relating to phosphorylation and methylation whilst dashed lines indicate protein diffusion. The labels +m and -m refer to the addition and removal of methyl groups to/from receptors, respectively. Similarly +p and -p represent the addition and removal of phosphoryl groups to/from chemotaxis proteins. Cell not drawn to scale.

model alongside a brief description of its derivation. In addition to this we mention the key assumptions underlying it and state the main simplifications considered in this work.

The spatial distribution of chemotaxis signalling proteins in *E. coli* cells is not generally considered to be significant in determining the chemotactic response. As such, it is possible to consider a mathematical model that assumes no spatial heterogeneity or any of the signalling proteins. This lack of spatial heterogeneity is one of the key assumptions underlying the law of mass action. As such Clausznitzer et al. [10] applied the law of mass action to reactions within the *E. coli* chemotaxis signalling cascade (see Figure S1), giving the non-linear ordinary differential equations (ODEs)

$$\frac{d[A_p]}{dt} = \Phi k_1([A_T]-[A_p]) - k_2[A_p]([Y_T]-[Y_p]) - k_3[A_p]([B_T]-[B_p]), \tag{S1}$$

$$\frac{d[Y_p]}{dt} = k_2[A_p]([Y_T]-[Y_p]) - k_4[Y_p][Z_T] - k_6[Y_p], \tag{S2}$$

$$\frac{d[B_P]}{dt} = k_3[A_p]([B_T]-[B_p]) - k_5[B_p], \tag{S3}$$

within which Φ denotes the receptor signalling team activity, k_i ($i = 1,2,3,4,5,6$) indicate the kinetic rates of each reaction and [...] denote the concentrations of the appropriate proteins with subscripts T and p indicating the total and phosphorylated amounts, respectively. It is worth noting here that the original model of Clausznitzer et al. [10] considers a separate model variable describing the binding of CheY-P with CheZ to form a transient complexed state ($[Y_pZ]$) and the subsequent dephosphorylation and unbinding. Due to the transient nature of this process, here it is assumed to occur rapidly enough to be modelled as a one step process. This allows it to be represented by a single term within equation (S2) (namely $-k_4[Y_p][Z_T]$).

In addition to the nonlinear ODE model of the phosphorylation cascade, Clausznitzer et al. [10]

Table S1: A base set of parameter values from the literature.

Symbol	Definition	Value	Source
$[A_T]$	Total concentration of CheA	7.9μM	[12]*
$[B_T]$	Total concentration of CheB	0.28μM	[12]*
$[R_T]$	Total concentration of CheR	0.16μM	[12]*
$[Y_T]$	Total concentration of CheY	9.7μM	[12]*
$[Z_T]$	Total concentration of CheZ	3.8μM	[12]*
k_1	CheA autophosphorylation	34s^{-1}	[13]
k_2	Phosphotransfer to CheY	100μM^{-1}s^{-1}	[14]
k_3	Phosphotransfer to CheB	15μM^{-1}s^{-1}	[14]
k_4	CheY-P dephosphorylation by CheZ	1.6μM^{-1}s^{-1}	[12]
k_5	Dephosphorylation of CheB-P	0.7s^{-1}	[15]
k_6	Dephosphorylation of CheY-P	0.085s^{-1}	[16]
g_R	Methylation by CheR	0.0375μM^{-1}s^{-1}	[10]
g_B	Demethylation by CheB-P	3.14μM^{-2}s^{-1}	[10]
N	Tar receptors in a signalling team	18	[17]
K_a^{on}	Dissociation constant: active Tar receptor	0.5mM	[18]$
K_a^{off}	Dissociation constant: inactive Tar receptor	0.02mM	[18]$
K_s^{on}	Dissociation constant: active Tsr receptor	1mM	[19]#
K_s^{off}	Dissociation constant: inactive Tsr receptor	0.0025mM	[19]#

* All total protein concentrations calculated from experimental values in [12] assuming a cellular volume of 1.4fl, as per [20].
$ Dissociation constant for Tar receptors to MeAsp.
\# Dissociation constant for Tsr receptors to serine.

produced a description of receptor methylation kinetics, as given by

$$\frac{dm}{dt} = g_R[R_T](1-\Phi) - g_B[B_p]^2\Phi, \tag{S4}$$

where m is the average methylation level of a receptor within the signalling team and g_R, g_B denote the kinetic rates of receptor methylation by CheR and demethylation by CheB-P, respectively. Further to this Clausznitzer et al. [10] define receptor signalling team activity (Φ) in equations (S1) and (S4) as a Monod-Wyman-Changeux (MWC) description of receptor clustering [11]. This is given by

$$\Phi = \frac{1}{1+e^F}, \tag{S5}$$

in which F, the free-energy of a receptor signalling team. Here we consider a slight alteration to the receptor free-energy expression considered in Clausznitzer et al. [10] which enables the consideration of two receptor types that independently sense separate ligands. This is defined by

$$F = N\left[h(m) + \nu_a \ln\left(\frac{1+[L_a]/K_a^{off}}{1+[L_a]/K_a^{on}}\right) + \nu_s \ln\left(\frac{1+[L_s]/K_s^{off}}{1+[L_s]/K_s^{on}}\right)\right], \tag{S6}$$

where N is the number of chemoreceptors in the signalling team, $1-m/2$ represents the offset energy (i.e. the contribution to F from the addition/removal of one methyl group) and the remainder of the expression is the free-energy contributions from Tar and Tsr receptors with dissociation constants for a single ligand denoted $K_{a/s}^{on/off}$ for active/inactive Tar (a) and Tsr (s) receptors and $[L]$ is the extracellular ligand concentration with a and s denoting the concentrations of aspartate and serine, respectively. Parameter values used in this model are listed in Table S1.

This mathematical model has been shown to be a good fit to experimental data in spite of the low copy numbers associated with CheR and CheB, suggesting that the continuum limit holds. With this being the case we do not anticipate stochastic effects would alter any conclusions drawn within this work.

The chemoreceptor signalling team free-energy expression in equation (S6) contains contributions of two different types of chemoreceptors for two different ligands (MeAsp and serine). It is known that both Tar and Tsr chemoreceptors may sense aspartate stimuli albeit with Tsr binding aspartate with a much lower affinity than Tar chemoreceptors. As such, it has been shown by Mello & Tu [21] that aspartate

binding to Tsr chemoreceptors has little or no effect at small to intermediate aspartate concentrations. As such, this effect has been neglected within this model. The work contained within the main manuscript describes simulations whereby either MeAsp is the sole attractant present or both MeAsp and serine are present simultaneously. In cases where both attratants are present we simply utilise equation (S6). However, where only MeAsp is present we may set $\nu_s = 0$ in order to remove the effect of Tsr receptors. This simplifies equation (S6) such that it becomes

$$F = N\left[h(m) + \ln\left(\frac{1+[L]/K_a^{off}}{1+[L]/K_a^{on}}\right)\right]. \tag{S7}$$

It is also worth noting here that Clausznitzer et al. [10] state the ratio of Tar to Tsr (ν_a:ν_s) receptors is equal to 1:1.4. As such, in this case, the symbol ν_a may be neglected since it is equal to one and thus will have no effect on the overall free-energy. The work of Clausznitzer et al. [10] compared this mathematical model to a range of experimental data. This demonstrated that the model represents a detailed enough description of the chemotaxis signalling pathway of *E. coli* cells as to adequately represent the biological processes involved.

References

[1] G. Wadhams and J. Armitage, "Making sense of it all: Bacterial chemotaxis," *Nature Reviews Molecular Cell Biology*, vol. 5, no. 12, pp. 1024–1037, 2004.

[2] J. Hess, K. Oosawa, N. Kaplan, and M. Simon, "Phosphorylation of three proteins in the signaling pathway of bacterial chemotaxis," *Cell*, vol. 53, no. 1, pp. 79–87, 1988.

[3] K. Lipkow, S. Andrews, and D. Bray, "Simulated diffusion of phosphorylated CheY through the cytoplasm of *Escherichia coli*," *Journal of Bacteriology*, vol. 187, no. 1, pp. 45–53, 2005.

[4] A. Bren, M. Welch, Y. Blat, and M. Eisenbach, "Signal termination in bacterial chemotaxis: CheZ mediates dephosphorylation of free rather than switch-bound CheY," *Proceedings of the National Academy of Sciences USA*, vol. 93, no. 19, pp. 10090–10093, 1996.

[5] M. Welch, K. Oosawa, S. Aizawa, and M. Eisenbach, "Phosphorylation-dependent binding of a signal molecule to the flagellar switch of bacteria," *Proceedings of the National Academy of Sciences USA*, vol. 90, no. 19, pp. 8787–8791, 1993.

[6] P. Cluzel, M. Surette, and S. Leibler, "An ultrasensitive bacterial motor revealed by monitoring signaling proteins in single cells," *Science*, vol. 287, no. 5458, pp. 1652–1655, 2000.

[7] K. Lipkow, "Changing cellular location of CheZ predicted by molecular simulations," *PLoS Computational Biology*, vol. 2, no. 4, p. e39, 2006.

[8] W. Springer and D. Koshland, "Identification of a protein methyltransferase as the CheR gene product in the bacterial sensing system," *Proceedings of the National Academy of Sciences USA*, vol. 74, no. 2, pp. 533–537, 1977.

[9] J. Stock and D. Koshland, "A protein methylesterase involved in bacterial sensing," *Proceedings of the National Academy of Sciences USA*, vol. 75, no. 8, pp. 3659–3663, 1978.

[10] D. Clausznitzer, O. Oleksiuk, L. Løvdok, V. Sourjik, and R. Endres, "Chemotactic response and adaptation dynamics in *Escherichia coli*," *PLoS Computational Biology*, vol. 6, no. 5, p. e1000784, 2010.

[11] J. Monod, J. Wyman, and J. Changeux, "On the nature of allosteric transitions: A plausible model," *Journal of Molecular Biology*, vol. 12, no. 1, pp. 88–118, 1965.

[12] M. Li and G. Hazelbauer, "Cellular stoichiometry of the components of the chemotaxis signaling complex," *Journal of Bacteriology*, vol. 186, no. 12, pp. 3687–3694, 2004.

[13] N. Francis, M. Levit, T. Shaikh, L. Melanson, J. Stock, and D. DeRosier, "Subunit organization in a soluble complex of Tar, CheW, and CheA by electron microscopy," *Journal of Biological Chemistry*, vol. 277, no. 39, pp. 36755–36759, 2002.

[14] R. Stewart, K. Jahreis, and J. Parkinson, "Rapid phosphotransfer to CheY from a CheA protein lacking the CheY-binding domain," *Biochemistry*, vol. 39, no. 43, pp. 13157–13165, 2000.

[15] R. Stewart, A. Roth, and F. Dahlquist, "Mutations that affect control of the methylesterase activity of CheB, a component of the chemotaxis adaptation system in *Escherichia coli*," *Journal of Bacteriology*, vol. 172, no. 6, pp. 3388–3399, 1990.

[16] J. Smith, J. Latiolais, G. Guanga, S. Citineni, R. Silversmith, and R. Bourret, "Investigation of the role of electrostatic charge in activation of the *Escherichia coli* response regulator CheY," *Journal of Bacteriology*, vol. 185, no. 21, pp. 6385–6391, 2003.

[17] R. Endres, O. Oleksiuk, C. Hansen, Y. Meir, V. Sourjik, and N. Wingreen, "Variable sizes of *Escherichia coli* chemoreceptor signaling teams," *Molecular Systems Biology*, vol. 4, no. 1, 2008.

[18] J. Keymer, R. Endres, M. Skoge, Y. Meir, and N. Wingreen, "Chemosensing in *Escherichia coli*: Two regimes of two-state receptors," *Proceedings of the National Academy of Sciences USA*, vol. 103, no. 6, pp. 1786–1791, 2006.

[19] C. Hansen, R. Endres, and N. Wingreen, "Chemotaxis in *Escherichia coli*: A molecular model for robust precise adaptation," *PLoS Computational Biology*, vol. 4, no. 1, p. e1, 2008.

[20] D. Bray, "Research group data." http://www.pdn.cam.ac.uk/groups/comp-cell/Data.html. Accessed: 03/06/2015.

[21] B. Mello and Y. Tu, "Effects of adaptation in maintaining high sensitivity over a wide range of backgrounds for *Escherichia coli* chemotaxis," *Biophysical Journal*, vol. 92, no. 7, pp. 2329–2337, 2007.
